# Quantitative proteomics reveals the antifungal effect of canthin-6-one isolated from *Ailanthus altissima* against *Fusarium oxysporum* f. sp. *cucumerinum in vitro*

**DOI:** 10.1371/journal.pone.0250712

**Published:** 2021-04-23

**Authors:** Yongchun Li, Meirong Zhao, Zhi Zhang

**Affiliations:** 1 School of Forestry, Northeast Forestry University, Harbin, China; 2 College of Chemistry and Life Science, Chifeng University, Chifeng, China; Fujian Agriculture and Forestry University, CHINA

## Abstract

Canthin-6-one, one of the main alkaloid compounds extracted from *Ailanthus altissima*, has recently attracted increasing interest for its antifungal activity. To evaluate the potential of canthin-6-one in controlling plant fungal diseases, we investigated the antifungal activity of canthin-6-one isolated from *A*. *altissima* against *Fusarium oxysporum* f. sp. *cucumerinum* (*Foc*) *in vitro*. The mycelial growth rate and micro-broth dilution were used to test antifungal activity. Furthermore, label-free quantitative proteomics and parallel reaction monitoring (PRM) techniques were applied to analyze the antifungal mechanism. It was found that canthin-6-one significantly inhibited the growth of *Foc*, and had higher inhibitory action than chlorothalonil at the same concentration. Proteomic analysis showed that the expression of 203 proteins altered significantly after canthin-6-one treatment. These differentially expressed proteins were mainly involved in amino acid biosynthesis and nitrogen metabolism pathways. These results suggest that canthin-6-one significantly interferes with the metabolism of amino acids. Therefore, it affects nitrogen nutrients and disturbs the normal physiological processes of fungi, and ultimately leads to the death of pathogens. This study provides a natural plant antifungal agent and a new perspective for the study of antifungal mechanisms.

## Introduction

*Ailanthus altissima* (Mill.) Swingle, belonging to the family Simaroubaceae, is a fast-growing deciduous tree that is native to Asia [1]. Phytochemical investigations have revealed that alkaloids, flavonoids, terpenoids, steroids, and many other biological components exist in *A*. *altissima* [2]. Among these compounds, alkaloids are the main and most common bioactive substances found in *A*. *altissima* [3]. Canthin-6-one, a subclass of β-carboline alkaloids with an additional D-ring, is one of the main alkaloids in *A*. *altissima* [4]. Many studies have shown that canthin-6-one possesses antiviral, anticancer, antimicrobial, and enzyme inhibitory properties [5]. In particular, the high antimicrobial activity of canthine-6-one has attracted extensive attention [6–8]. However, most of these studies have been associated with the medical field, and only a few studies have reported the effects of this compound on plant pathogens.

Plant diseases, particularly fungal diseases, can cause crop losses and exert serious biological pressure on food security [9, 10]. *Fusarium* is one of the most serious pathogenic fungi and can cause wilt, rot, and canker diseases in many plants [11, 12]. At present, chemical sterilization is the main control method for *Fusarium* disease. However, this method causes a series of problems, including chemical resistance, chemical residues, and environmental and health hazards [13]. Secondary metabolites isolated from plants have recently attracted increasing interest for their antifungal activity. The compounds derived from plants generally have the characteristics of biodegradation and low toxicity residues, which are safer to human health than synthetic chemicals [14]. In a preliminary study, we found that canthin-6-one isolated from *A*. *altissima* exhibited antifungal activity against some plant pathogenic fungi, such as *F*. *graminearum*, *F*. *oxysporum* f. sp. *benincasae*, *F*. *oxysporum* f. sp. *cucumerinum* (*Foc*), and *Gloeosporium* sp. Nevertheless, the antifungal activity and mechanism of canthin-6-one as a fungicide remain unclear. In this study, *Foc* was used as the fungal strain to investigate the inhibitory effect of canthin-6-one on mycelial growth. In addition, proteomics techniques have been used to determine the pathway of action of canthin-6-one.

## Materials and methods

### Preparation of canthin-6-one

Canthin-6-one was extracted and isolated from the stem bark of *A*. *altissima*, which was purchased from Jiaxu Pharmaceutical Co., Ltd., Bozhou City, Anhui Province, China, in June 2017. The specimen (No. 2017-AA02) was deposited in the Laboratory of Plant Resources, College of Chemistry and Life Science, Chifeng University, Inner Mongolia, China. The extraction and isolation of canthin-6-one was carried out using previously published methods [3]. The structure of canthin-6-one was determined using physical (m. p.) and spectral analyses (^13^C-NMR and ^1^H-NMR). The purity of canthin-6-one was evaluated using high-performance liquid chromatography (HPLC), and found to have a purity greater than 98%. Canthin-6-one was then dissolved in dimethyl sulfoxide (DMSO, 1% v/v).

### Culture of fungal mycelia and preparation of spores

The fungal strain of *Foc* (No. 2532) was obtained from the China Center of Industrial Culture Collection (CICC), Beijing, China. The strain was inoculated in potato dextrose agar (PDA) medium (6 g L^-1^ potato extract, 20 g L^-1^ glucose, and 20 g L^-1^ agar) and cultured at 25°C for 6 days. Mycelia were used to prepare spore suspensions and to set up the antifungal activity assays. Mycelium disks (6 mm in diameter) were cut from 6-day-old cultures and suspended in potato dextrose broth (PDB) medium (6 g L^-1^ potato extract, 20 g L^-1^ glucose). After oscillation, filtration, and microscopic examination, the concentration of the suspension was maintained at approximately 1 × 10^6^ spores mL^-1^.

### Evaluation of the antifungal activity

#### MIC assay

The minimal inhibitory concentration (MIC) was determined using the microdilution method [15–17]. The canthin-6-one samples were diluted with PDB to prepare stock solutions of different concentrations. The stock solution (50 μL) and spore suspension (50 μL) were dispensed into each well of 96-well plates, and the canthin-6-one concentrations were adjusted to 1.0, 2.0, 4.0, 8.0, 16.0, 32.0, 64.0, and 128.0 μg mL^-1^. Wells without canthin-6-one were used as the blank controls. The plates were covered and incubated at 25°C. The MIC was defined as the lowest concentration at which no visual growth of fungal mycelium was observed when the fungal mycelium appeared in the control medium [18–20].

#### Inhibitory activity assay

Inhibitory activity against the fungal mycelium was tested using a previously described protocol with a few modifications [14]. PDA medium plates (90 mm in diameter) containing canthin-6-one at different concentrations (1.0, 2.0, 4.0, 8.0, and 16.0 μg mL^-1^) were prepared. Equal amounts of chlorothalonil and DMSO were added to PDA medium as a positive control and blank control, respectively. One mycelium disk (6 mm in diameter) was cut from the edge of 6-day-old mycelia cultures, placed in the center of each PDA plate, and incubated upside down at 25°C for 72 h. Each test was performed in triplicate. The crisscross method was used to measure the diameters of the fungal colonies. The mycelium growth inhibitory rate (%) was determined as follows: I (%) = (C—T)/(C—K) × 100, where C and T are the average diameters of the fungal colonies in the control and treatment, respectively, and K is the average diameter of the inoculum disk. Then, the inhibitory rate-dose response curve and linear regression equation were obtained. The effective concentration for 50% inhibition (EC_50_) was calculated using the regression equation [21].

#### Observation of hyphal morphology

The spore suspension was cultured in PDB medium at 25°C. After 48 h, canthin-6-one was added to the suspension. The final concentrations of canthin-6-one were 0, 8.0, 16.0, 32.0, 64.0, and 128.0 μg mL^-1^. The mixed solution was then incubated at 25°C for 48 h. The resulting mycelia were collected, cleaned with PBS (0.1 M, pH 7.0), and observed under a light microscope (Olympus BX53F, Olympus, Tokyo, Japan) at 400× magnification.

### Stability test of antifungal activity

The canthin-6-one solutions (4.2 μg ml^-1^, EC_50_) were treated with ultraviolet radiation, acid-base treatment, and varying temperatures and storage time. The inhibition rate of mycelial growth was used to investigate the antimicrobial stability of canthin-6-one after different treatments. Ultraviolet irradiation treatment was carried out with a 30 W ultraviolet lamp for 0, 3, 6, 12, and 24 h. Temperature treatment was performed in a refrigerator (4°C), water bath (25°C, 50°C, 100°C), and a high-temperature sterilizing oven (121°C) for 1 h. The pH of the PDA medium containing canthin-6-one was adjusted to pH 3, 4, 5, 6, 7, 8, 9, and 10 with sodium hydroxide or hydrochloric acid, and the PDA medium containing DMSO with the same acid base was used as a control. The storage conditions were as follows: canthin-6-one solutions were stored in the dark at 25°C for 30, 60, 90, 120, and 180 d. The mycelium growth inhibitory rate was determined according to the method of inhibition activity assay.

### Quantitative proteomics experiments

#### Protein extraction and digestion

The spore suspension was cultured in PDB medium at 25°C. After 24 h, a certain amount of canthin-6-one was added to the suspension, and the final concentration of canthin-6-one was 4.2 μg mL^-1^ (EC_50_). An equal amount of DMSO was added as a blank control. The mixed solution was then incubated at 25°C for 24 h. The mycelia were collected and washed three times with 1× PBS for protein extraction. Three biological replicates were used for each sample. Protein extraction and digestion were performed according to a previously described method [22]. Proteins were extracted using SDT buffer (4% sodium dodecyl sulfate, 0.1 M dithiothreitol, 100 mM Tris/HCl, pH 7.6), and then quantified using the BCA protein assay reagent (Bio-Rad Laboratories, Inc., Hercules, CA, USA). The filter-aided proteome preparation (FASP) method [23] was used to trypsinize (Promega, Madison, WI, USA), the appropriate amount of protein from each sample, and then the C18 cartridge (Empore™ SPE, bed I.D. 7 mm, volume 3 mL; Sigma, Kawasaki, Japan) was used to desalinize the hydrolyzed peptide. Dissolution buffer (40 μL, 0.1% trifluoroacetic acid) was added to the lyophilized peptides for reconstitution, and the peptide content was estimated using UV spectroscopy at 280 nm.

#### Liquid chromatography-tandem mass spectrometry (LC-MS/MS) analysis

LC-MS/MS experiments were performed on a Q Exactive mass spectrometer coupled to an Easy nLC system (Thermo Fisher Scientific, Waltham, MA, USA), and the procedure was performed according to Li et al. [22]. First, the peptides of each sample were injected into a C_18_ column (Easy, 10 cm long, 75 μm I.D., 3 μm resin; Thermo Scientific) in buffer A (0.1% formic acid), and then separated with buffer B (0.1% formic acid and 84% acetonitrile) at a flow rate of 250 nL min^-1^. The solvent gradient was as follows: 0–35% buffer B for 50 min, 35–100% buffer B for 8 min, and then 100% buffer B for 3 min. MS analysis was performed using Q Exactive (Thermo Fisher Scientific). The survey scan range of precursor ions was 300–1800 m/z, and the 10 most abundant polypeptide fragments were dynamically selected. The duration of dynamic exclusion was 40.0 s. The target value was determined using an automatic gain control (AGC). Survey scans were obtained at a resolution of 70,000 at m/z 200, and the resolution for the HCD spectra was 17,500 at m/z 200. The normalized collision energy was 30 eV and the underfill ratio was 0.1%. The MS data were compared with the UniProt *Fusarium oxysporum* database (342953 sequences, downloaded May 2019) using MaxQuant software (v.1.5.5.1). Trypsin was used as a cleavage enzyme, allowing a maximum of two missing cleavages. The peptide mass tolerance was set to 20 ppm, and the main search used a mass window of 6 ppm. Carbamidomethylation of cysteine and oxidation of methionine were defined as fixed and variable modifications, respectively. The false discovery rate (FDR) for peptide and protein identification was set at ≤0.01.

#### Bioinformatics methods

Gene ontology (GO) annotations and Kyoto Encyclopedia of Genes and Genomes (KEGG) pathway analysis were performed using the software Omicsbean (http://www.omicsbean.cn/) to identify differentially expressed proteins [24]. Protein-protein interaction (PPI) networks were analyzed using the STRING database (https://string-db.org/). Cytoscape open-source software (https://cytoscape.org/) was used for visualization, and the orphan protein was deleted.

#### Parallel Reaction Monitoring (PRM) analysis

Additional quantification was performed by liquid chromatography parallel reaction monitoring mass spectrometry (LC-PRM/MS) to further check the changes in protein expression. Protein extraction and digestion were performed according to the procedure described in “Protein extraction and digestion”. The PRM analysis was performed using a Q Exactive mass spectrometer and an Easy nLc system (Thermo Fisher Scientific, Bremen, Germany). The digested peptide and iRT-Kit peptide (Biognosys AG, Zurich, Switzerland) were mixed in equal amounts as an internal standard. Each sample was detected by LC-PRM/MS, and PRM data analysis was performed using the Skyline software (3.5.0) [22]. Significant differences were determined by Student’s *t*-test at a significance level of *p* < 0.05.

## Results

### Inhibition of canthin-6-one against *Fusarium oxysporium* f. sp. *cucumerinum*

**Fig 1:**
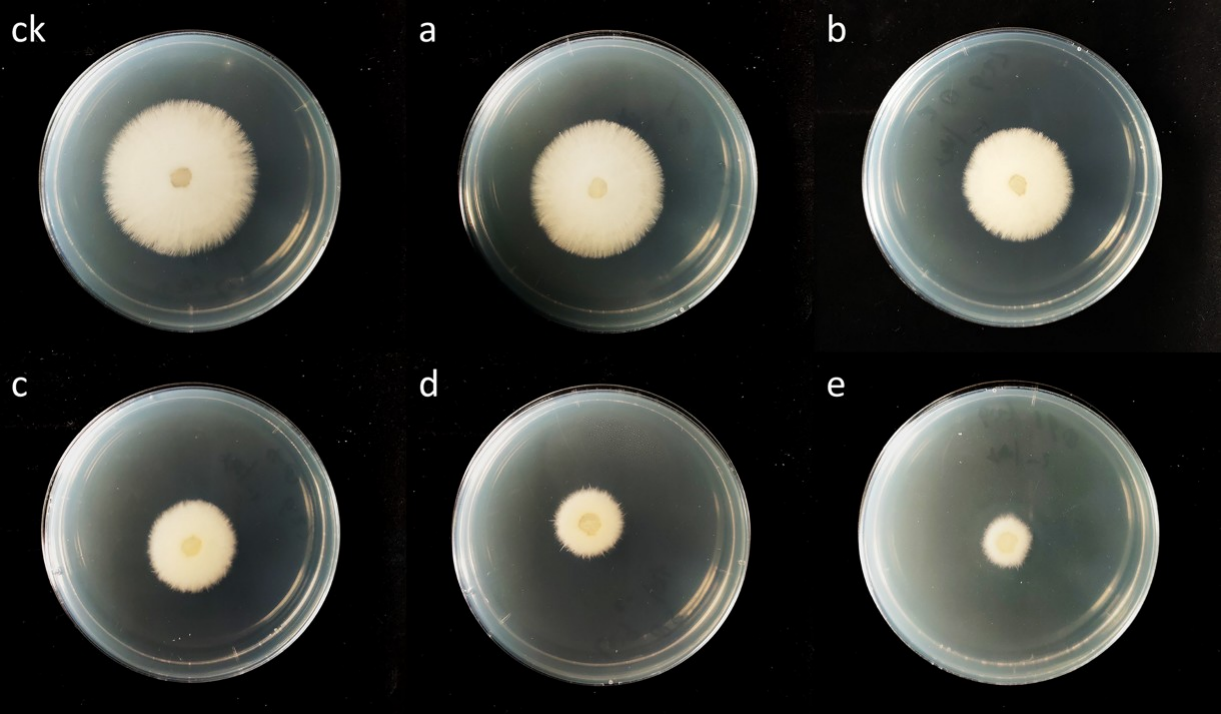
Inhibition effect of canthin-6-one on mycelium growth (72 h). ck, blank control; a–e, treatment with 1.0, 2.0, 4.0, 8.0, 16.0 μg mL^-1^ canthin-6-one, respectively.

The MIC was used to quantitatively evaluate the antifungal activity. In this study, the MIC value of canthin-6-one against *Foc* was detected through fungal mycelium growth or no growth, with a value of 32.0 μg mL^-1^. The results showed that canthin-6-one exhibited strong antifungal activity against the tested fungal strains. The inhibitory effect of canthin-6-one on mycelial growth *in vitro* was concentration-dependent. As shown in Fig 1, the higher the concentration of canthin-6-one, the more obvious the inhibition of mycelial growth.

The inhibition rate reached over 81% at the highest concentration of canthin-6-one (16.0 μg mL^-1^), which was clearly superior to that of chlorothalonil (40.1%) (Table 1). The EC_50_ value calculated by the regression equation was approximately 4.2 μg mL^-1^.

**Table 1:** Effect of canthin-6-one on mycelium growth.

Treatment	Concentration (μg mL^-1^)	Colony diameter (mm)	Inhibition rate (%)
**Canthin-6-one**	0.0	39.2 ± 0.2^a^	
	1.0	33.2 ± 0.3^b^	15.5 ± 0.94^f^
	2.0	26.4 ± 0.6^c^	32.8 ± 1.42^e^
	4.0	20.5 ± 0.5^e^	47.9 ± 0.98^c^
	8.0	13.0 ± 0.2^f^	66.8 ± 0.65^b^
	16.0	7.3 ± 0.2^g^	81.5 ± 0.61^a^
**Chlorothalonil**	16.0	22.9 ± 0.6^d^	40.1 ± 1.55^d^

Note: The values are the mean of three replicates ± SD (n = 3). Different letters after means indicate significant differences at *p* < 0.05.

**Fig 2:**
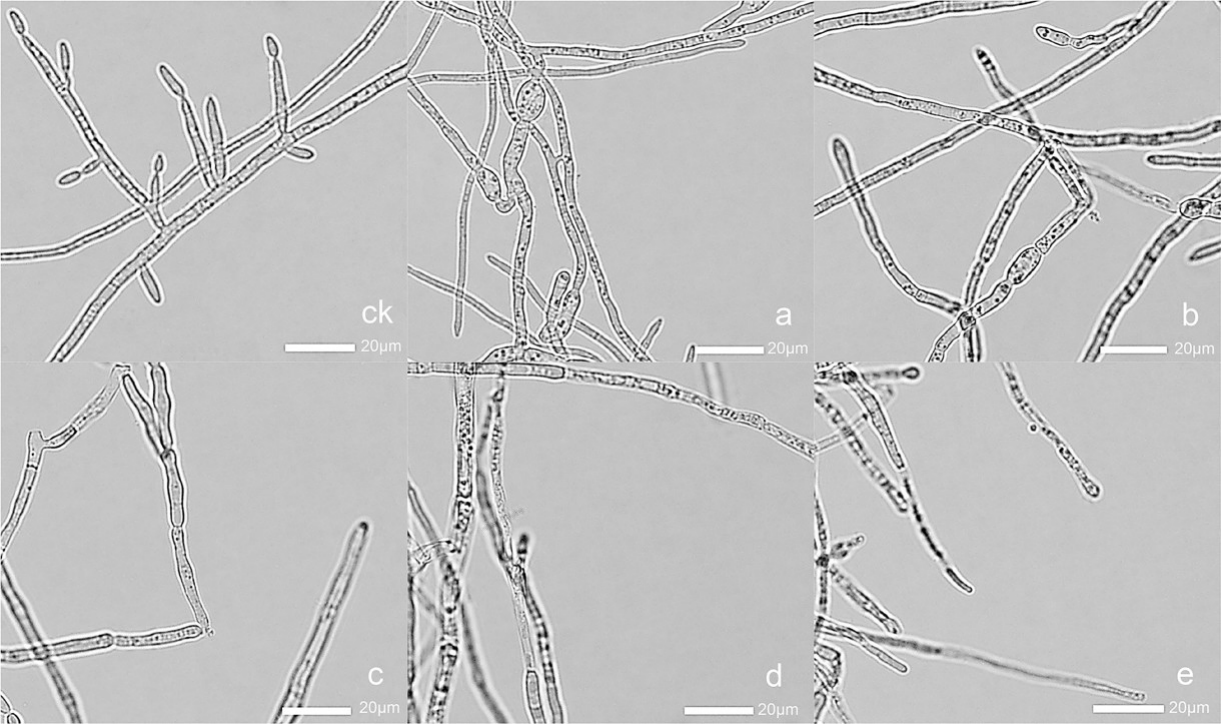
The effect of canthin-6-one on hyphal morphology. ck, blank control; a–e, treatment with 8.0, 16.0, 32.0, 64.0, 128.0 μg mL^-1^ canthin-6-one, respectively.

Hyphal morphology was observed using a light microscope at 400× magnification. Compared with the control, the fungal hyphal grown in PDB containing alkaloids showed alterations in morphology (Fig 2). After treatment with canthin-6-one, the hyphal became thinner, branching decreased, and spore formation was reduced. At the highest concentration (128.0 μg mL^-1^), the morphology of hyphal changed significantly. These results suggested that canthin-6-one significantly reduced the hyphal branching, and destroyed the morphology of the hyphal.

### Analysis of the antifungal stability of canthin-6-one

**Fig 3:**
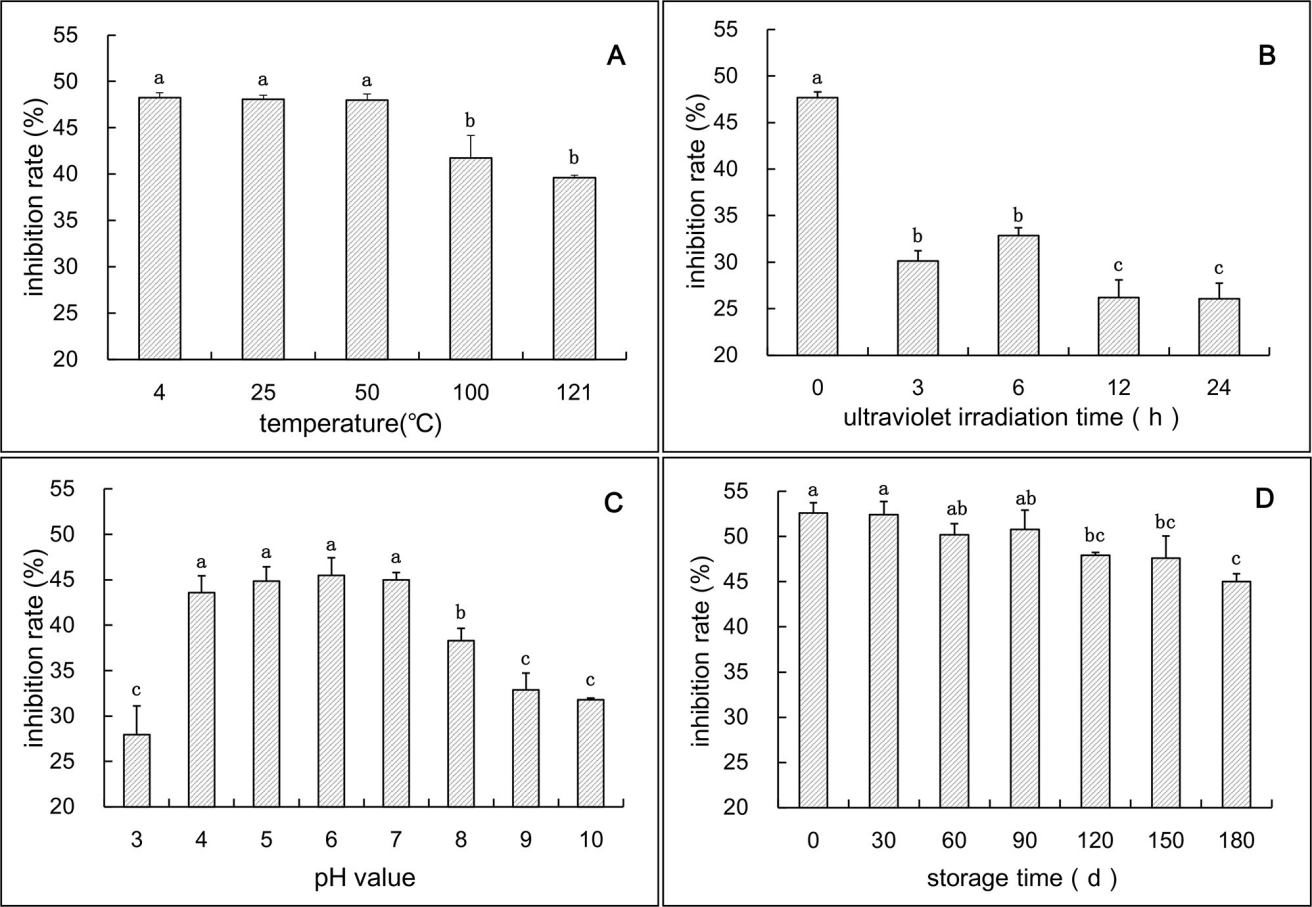
Antifungal activity of canthin-6-one alkaloid treated by different factors. A, temperature treatment; B, ultraviolet irradiation treatment; C, acid or alkali treatment; D, storage time. Different letters indicate significant differences (*p <* 0.05).

The antimicrobial ability of active compounds is usually affected by certain conditions, such as temperature, ultraviolet radiation, pH, and storage time. In this study, the mycelium growth inhibitory rate of canthin-6-one treated with different external factors was determined. The results showed that the antifungal activity of canthin-6-one was not significantly altered at lower temperatures (4°C, 25°C, and 50°C), and the inhibition rates were 48.27%, 48.07%, and 47.97%, respectively (Fig 3A). After high-temperature treatment (100°C and 121°C), the antifungal activity decreased to 41.75% and 39.61%, respectively, indicating that canthin-6-one was thermally unstable and high-temperature treatment should be avoided. As shown in Fig 3B, the antifungal activity of canthin-6-one decreased after UV irradiation treatment (*p* < 0.05). Compared with the control, the mycelial growth inhibition rate decreased by 37–45% after 3–24 h of UV irradiation. Under different pH conditions, canthin-6-one showed different antifungal activity. In this study, the effect of acid or alkali treatment on the antifungal activity of this compound was analyzed. The results showed that the antifungal ability of canthin-6-one was significantly obvious under the condition of pH 4–7 compared with pH 3 or pH 8–10 (*p* < 0.05), and the mycelial growth inhibition rate reached 43.57–45.5% (Fig 3C). The validity period is an important indicator of antifungal agents, and the effect of storage time on the antifungal activity of canthin-6-one was analyzed. The results indicated that the mycelium growth inhibition rate did not change significantly between 0–3 months. In the 3–6 month storage period, the inhibition rate decreased slightly (Fig 3D).

### Quantitative proteomics analysis

#### Changes in *Foc* protein expression after canthin-6-one treatment

Quantitative proteomics analysis was carried out using a label-free approach to elucidate the possible antifungal mechanism of canthin-6-one against *Foc*. Based on the database of *Fusarium oxysporum*, 1389 proteins with quantitative information were identified. The expression of 1186 proteins (85.4%) of mycelia did not show obvious differences between the treatment (canthin-6-one) and control (ck) groups. Compared with the control groups, the expression of 203 proteins in treatment groups changed, 123 of which were down-regulated, and 80 of them were up-regulated (fold changes > 1.5, *p* < 0.05) (S1 Fig, S1 Table). The screening of these differentially expressed proteins was reasonable and could effectively distinguish the comparison groups according to the hierarchical clustering heatmap (S2 Fig). These results indicated that canthin-6-one stimulated physiological responses and induced different expression of some proteins in the tested fungal strain.

#### Bioinformatics analysis of differentially expressed proteins

The properties and functions of these differentially expressed proteins were elucidated using bioinformatics analyses (S2 Table). GO annotations were performed in biological process (BP), cellular component (CC), and molecular function (MF) [25, 26]. According to the BP, these proteins were mainly involved in the organic acid metabolic process, oxoacid metabolic process, small molecule metabolic process, carboxylic acid metabolic process, and organonitrogen compound metabolic process. CC analysis showed that masses of proteins were localized intracellularly, mainly in the cytoplasm. Meanwhile, a large number of these proteins participated in pyridoxal phosphate binding, transferase activity, transferring glycosyl groups, cofactor binding, and antioxidant activity, according to the analysis of MF (S3 Fig).

**Fig 4:**
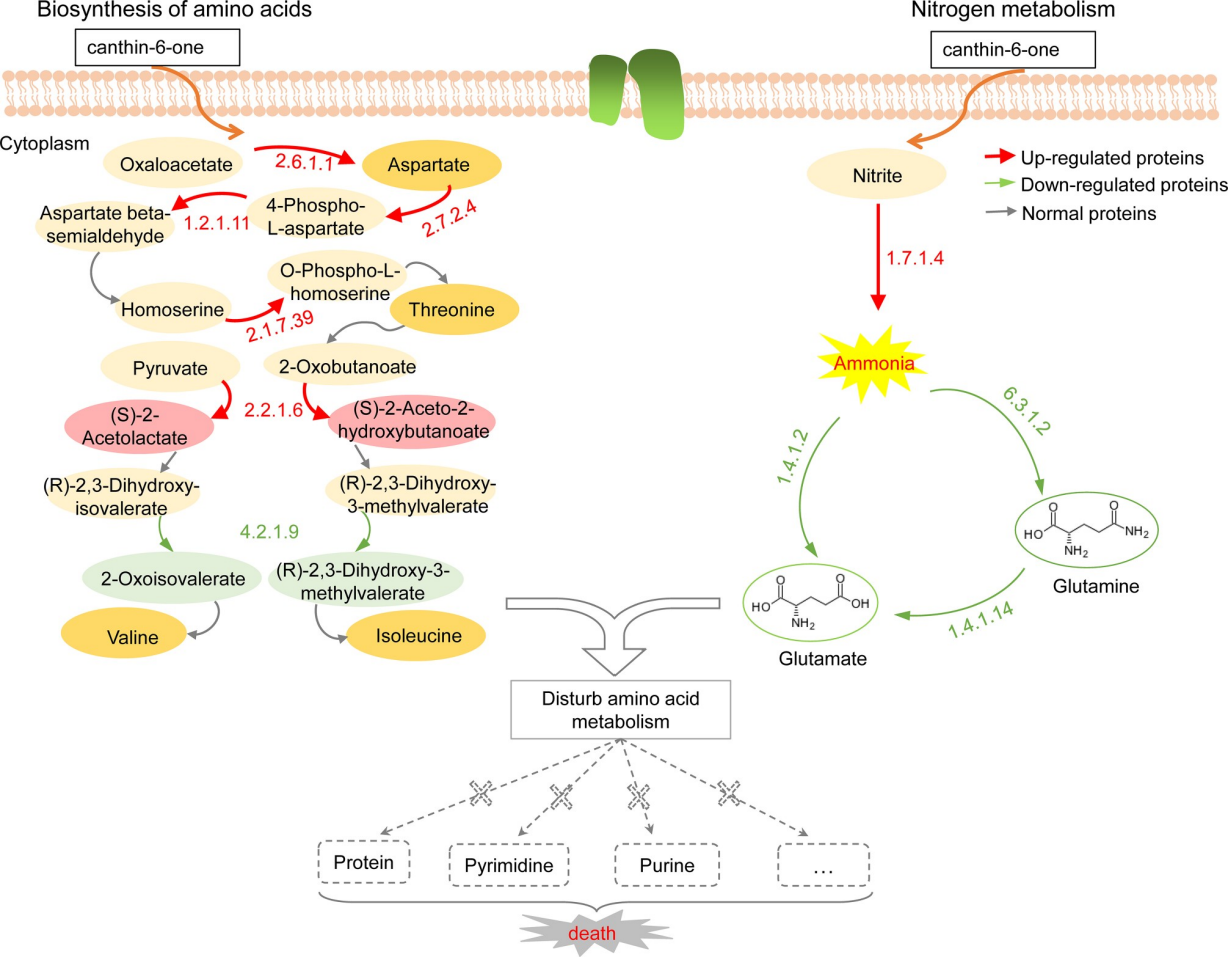
Schematic of amino acid biosynthesis and nitrogen metabolism pathways in response to canthin-6-one.

The KEGG database was used to identify the pathways involved in differentially expressed proteins [27]. Through KEGG pathway analysis, two main metabolic pathways, amino acid biosynthesis and nitrogen metabolism pathways, were locked (S4 Fig). These pathways are known to play a significant role in amino acid metabolism. The detailed pathway is shown in Fig 4. In the pathway of amino acid biosynthesis, the expression of many enzymes was changed, upregulated, or downregulated, which could affect normal amino acid metabolism. For example, the expression of acetolactate synthase (EC 2.2.1.6) was upregulated, which greatly increased the biosynthesis of (S)-2-Acetolactate and (S)-2-Aceto-2-hydroxybutanoate. However, in the subsequent step, the downregulated expression of dihydroxyacid dehydratase (EC 4.2.1.9) significantly inhibited the normal synthesis of the intermediates 2-Oxoisovalerate and (S)-3-Methyl-2-oxopentanoate, which resulted in the blockage of this synthetic route and the shortage of valine and isoleucine. Similarly, overexpressed proteins were also found in threonine synthesis, potentially causing imbalances in these intermediate metabolites and further disrupting normal metabolic pathways. In another pathway, nitrogen metabolism is disturbed by the expression of some enzymes. The expression of nitrite reductase (EC 1.7.1.4) was upregulated, but glutamate synthetase (EC 1.4.1.14), glutamate dehydrogenase (EC 1.4.1.2), and glutamine synthetase (EC 6.3.1.2) were downregulated in subsequent steps. The change in the expression of these enzymes may lead to an excessive accumulation of ammonia and a reduction of glutamate and glutamine. Amino acids are key materials for the synthesis of proteins, nucleic acids, phospholipids, and vitamins, among others. Ammonia is an important substance in nitrogen nutrition, but excessive ammonia is harmful to mycelial growth. The reduction of glutamate and glutamine directly disrupts the synthesis of purine and pyrimidine, which further affects nucleic acid metabolism. Thus, canthin-6-one could significantly regulate the expression of some critical enzymes, which disturbed the normal amino acid metabolism process. These consequences might further damage the metabolism of proteins, nucleic acids, or other substances, resulting in the death of pathogens.

**Fig 5:**
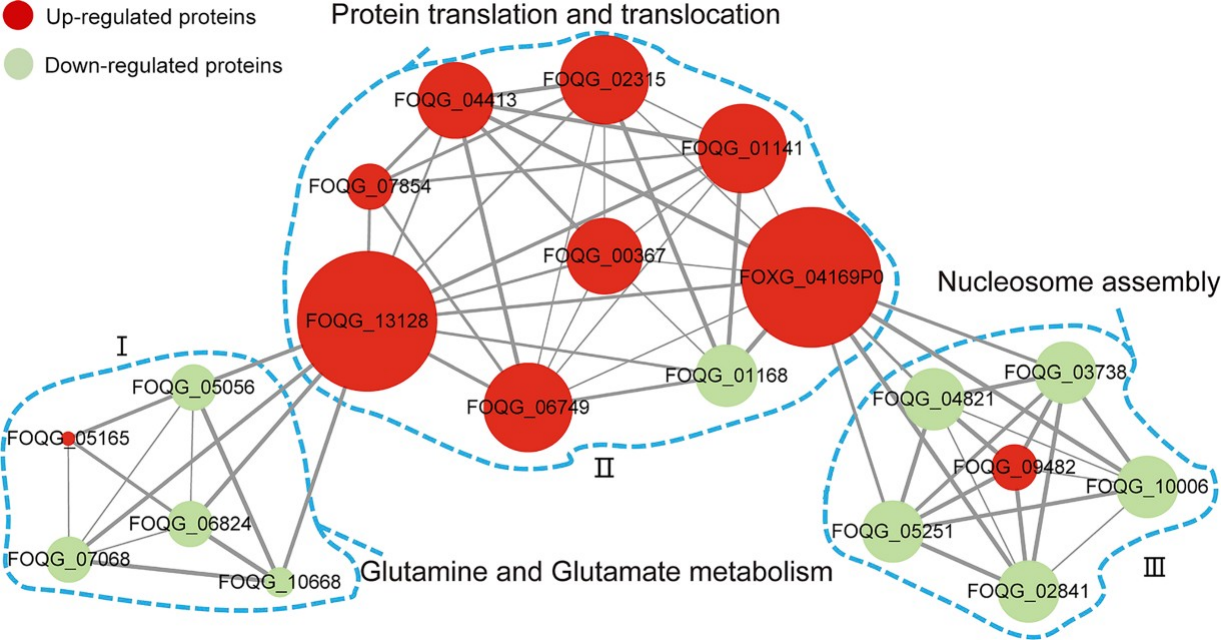
Analysis of protein-protein interaction network for differentially expressed proteins. The nodes represent proteins (down-regulated and up-regulated), and edges represent interaction between proteins. Node size and edge width indicate the number of connected nodes and the strength of interaction, respectively.

To further understand the functions and associations of these differentially expressed proteins, protein-protein interaction networks were established using the STRING database [28]. In this section, the 20 highest-scoring differentially expressed proteins with strong interactions were screened for interaction network analysis. As shown in Fig 5, the proteins were divided into three different clusters. The connections of Part I indicated that the proteins involved in amino acid biosynthesis were significantly disturbed. The process of protein translation and post-translational translocation was also seriously affected in Part II. In addition, the connections of Part III indicated that proteins involved in nucleosome assembly might be triggered by protein folding and degradation systems, which affect the nucleic acid metabolism pathway. These interactions suggest that canthin-6-one may significantly affect various physiological processes and cause pathogen death.

### Quantitative analysis of protein by PRM

**Fig 6:**
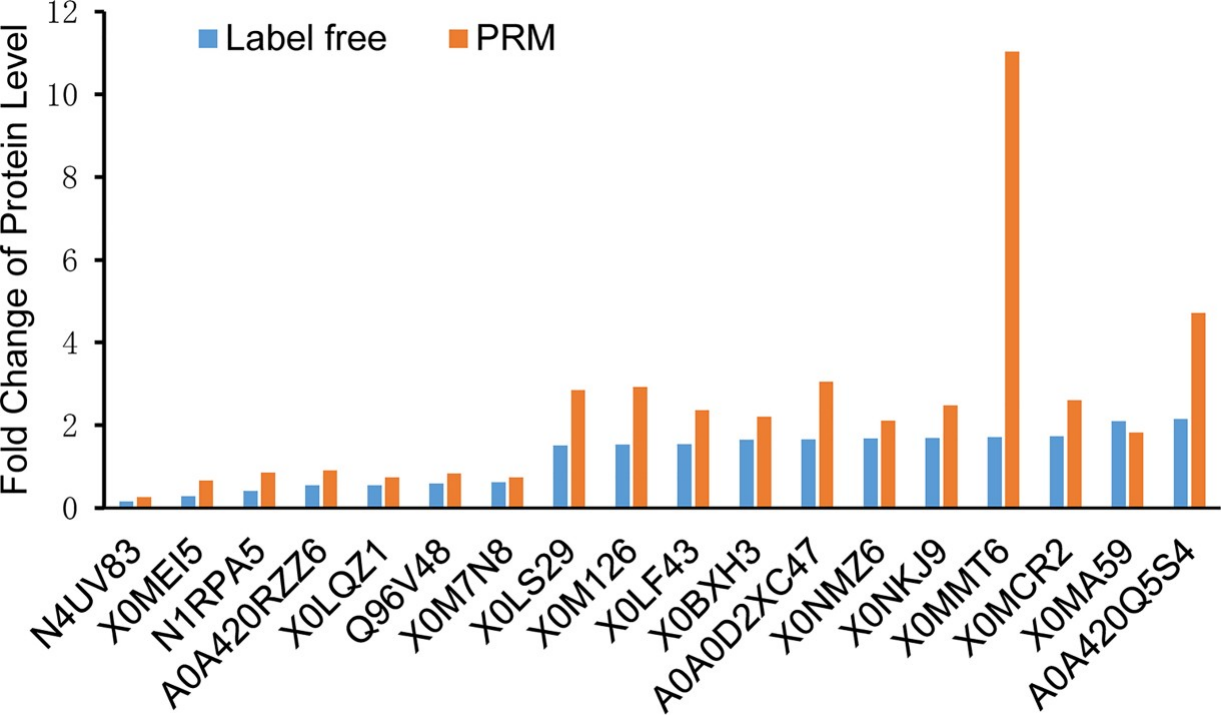
PRM analysis for 18 selected differentially expressed proteins.

The detection of related gene transcription and PRM are considered the two main methods to further evaluate the expression levels of target proteins [29]. Compared with gene transcription detection, the PRM technique is more accurate and comprehensive in quantitative protein analysis [30–33]. Therefore, PRM was used to further verify the results of label-free quantitative proteomics in this study. According to the results of the bioinformatics analysis, 18 differentially expressed proteins were selected for PRM analysis (S3 Table). The fold change of protein levels analyzed using PRM and label-free quantitative proteomics is shown in Fig 6. The results showed that the tendency of 18 proteins identified using PRM was similar to the label-free quantitative proteomics data, demonstrating that the label-free results were reliable.

## Discussion

Plants are an important biological resource and contain many compounds that play an important role in metabolic disorders [34]. Owing to their biodegradability and low toxicity, plant-derived compounds are widely used in the development of biological pesticides. *A*. *altissima* is a resourceful tree that contains many bioactive substances. Canthin-6-one is an important bioactive substance in *A*. *altissima*, and its antifungal ability is worthy of attention. In this study, the antifungal activity and mechanism of canthin-6-one isolated from *A*. *altissima* against *Foc* were evaluated.

### Canthin-6-one from *A*. *altissima* inhibits the growth of *Foc*

Antifungal activity analysis showed that canthin-6-one is able to inhibit the growth of *Foc* pathogen, with an MIC value of 32 μg mL^-1^. The level of antifungal activity observed here is similar to that reported for canthin-6-one isolated from *Zanthoxylum chiloperone* var. *angustifolium* against *Candida albicans*, *Aspergillus fumigatus*, and *Trichophyton mentagrophytes* var. *interdigitale* [35]. Mycelium growth is an important indicator of fungi. In this study, we analyzed the effect of canthin-6-one on the mycelial growth and morphology of *Foc*. The results indicated that canthin-6-one could significantly inhibit the growth and damage the structure of *Foc* (Table 1, Fig 2). It was also found that the inhibition rate of canthin-6-one was superior to chlorothalonil at the same concentration. Based on these preliminary results, canthin-6-one may be a natural antifungal candidate for further exploration and development in future studies.

### The antifungal activity of canthin-6-one from *A*. *altissima* is relatively stable

Antifungal stability analysis (Fig 3) showed that canthin-6-one was relatively stable. The stability of antifungal activity at 4–50°C (Fig 3A) suggested that canthin-6-one has a wide range of temperature applications and is not affected by temperature in agricultural production. In neutral and acidic environments, canthin-6-one maintained a high antifungal activity (Fig 3C). In recent years, soil acidification has become an increasingly serious issue owing to the influence of acid rain and chemical fertilizers. Under acidic soil conditions, canthin-6-one may be advantageous as an antifungal agent. In addition, the antifungal activity of canthin-6-one decreased slightly in the dark at 25°C within 6 months (Fig 3D). In contrast, canthin-6-one was sensitive to ultraviolet irradiation (Fig 3B). Therefore, canthin-6-one needs to be stored away from light. The degradability of canthin-6-one under ultraviolet light is beneficial for its application in eco-friendly agriculture.

### Canthin-6-one from *A*. *altissima* disturbs amino acid biosynthesis and nitrogen metabolism of *Foc*

Understanding the antifungal mechanism of canthin-6-one is key for the development and utilization of antifungal agents. The mechanism of action of canthin-6-one against *Saccharomyces cerevisiae* was previously reported to affect fatty acid metabolism by stimulating desaturase enzyme systems [7]. Further research has shown that canthin-6-one can permeate the cell membrane by binding to the sterol part of the membrane of *S*. *cerevisiae*, causing the death of the fungus [36]. These studies partially explained the antifungal mechanism of canthin-6-one. In recent years, quantitative proteomics has become a useful approach for revealing antimicrobial mechanisms [28, 37]. The combination of label-free quantitative proteomics and PRM-based targeted mass spectrometry is a sensitive and accurate method for protein quantification [22, 37].

In this study, label-free quantitative proteomics was applied to investigate the antifungal effect of canthin-6-one against *Foc* at the proteome level. The protein expression of two groups of samples, the control and samples treated with canthin-6-one, were detected by LC-MS/MS. The results showed that 203 proteins were differentially expressed between the two groups, indicating that canthin-6-one affected the expression of proteins. Through GO and KEGG bioinformatics analysis, these differentially expressed proteins were found to be mainly involved in amino acid biosynthesis and nitrogen metabolism pathways. Among these metabolic pathways, glutamine synthetase is considered the central and key enzyme for nitrogen assimilation and glutamine biosynthesis [38]. The expression of glutamine synthetase (EC 6.3.1.2) was significantly downregulated after canthin-6-one treatment. The downregulated expression of this enzyme disrupted the synthesis of glutamate and glutamine (Fig 4), and may further affect the metabolism of other amino acids, nucleic acids, or other substances. In addition, it was found that amino acid transporters, such as dicarboxylic acid amino acid permease, were strongly downregulated, which has the potential to interfere with the transport of amino acids. Protein-protein interaction analysis of these differentially expressed proteins also showed that canthin-6-one disrupted multiple physiological processes, amino acid metabolism, protein translation and post-translational translocation, and nucleosome assembly (Fig 5). Normal metabolism is essential for mycelial growth; however, the blockage or disturbance of the metabolic pathway can cause a series of problems, such as biochemical disorders, growth inhibition, and cell death. Some studies have shown that amino acids are the main source of nutrients for fungi, and their metabolic pathways and transported elements could be used as targets for antifungal drugs [39, 40]. Therefore, the metabolic process of amino acids is blocked, which can be fatal to the nutrition and growth of fungi. In this study, the synthesis of branched-chain amino acids was also found to be severely disturbed in the amino acid biosynthesis pathway, providing an insight into potential antifungal targets of canthin-6-one.

In summary, canthin-6-one effectively inhibited the growth of *Foc*, further confirming its antifungal potential against plant pathogens. Proteomic analysis revealed that canthin-6-one could significantly interfere with the metabolism of amino acids and affect the fungal nutrients, resulting in the disorder of normal physiological processes and the death of pathogens. Thus, amino acid metabolic pathways and transported elements may be targets for canthin-6-one action. This study provides an insight into the molecular mechanism of canthin-6-one against plant pathogens and provides a natural antifungal agent candidate derived from plants for the protection of plants during agricultural production. Nevertheless, more studies are still needed to evaluate the potential use of canthin-6-one as an antifungal agent and to elucidate the complete mechanism of its antifungal action.

## Supporting information

S1 Fig Venn diagrams (a) and Volcano plot (b) of expressed proteins between control and treatment groups.
(TIF)

S2 Fig Hierarchical clustering heatmap of differentially expressed proteins in the comparison groups (s/ck) (fold > 1.5, *p* < 0.05). The relative abundance of proteins is represented by different colors, where red represents higher intensity and blue represents lower intensity.
(TIF)

S3 Fig GO analysis of biological process (a), cellular component (b), and molecular function (c).
(TIF)

S4 Fig KEGG pathway enrichment bubble plot of differentially expressed proteins. The vertical axis represents the enriched KEGG classification (fold > 1.5, *p* < 0.05). The horizontal axis is the rich factor (rich factor ≤ 1), representing the ratio of the number of differentially expressed proteins to those identified in the KEGG pathway. The size of the circular area represents the number of differentially expressed proteins, and the circular color represents the enrichment p-value of the differentially expressed proteins under the KEGG classification.
(TIF)

S1 Table Details of 203 differentially expressed proteins. (XLSX)

S2 Table Bioinformatics analysis of differentially expressed proteins. (XLSX)

S3 Table Comparison of PRM analysis with the label-free quantitative proteomics triggered by canthin-6-one. (XLSX)
