# Supplementary figures and images for: Quantitative proteomics reveals the antifungal effect of canthin-6-one isolated from Ailanthus altissima against Fusarium oxysporum f. sp. cucumerinum in vitro
Source: PLoS One. 2021 Apr 23;16(4):e0250712. doi: 10.1371/journal.pone.0250712 (PMC8064541; doi:10.1371/journal.pone.0250712)

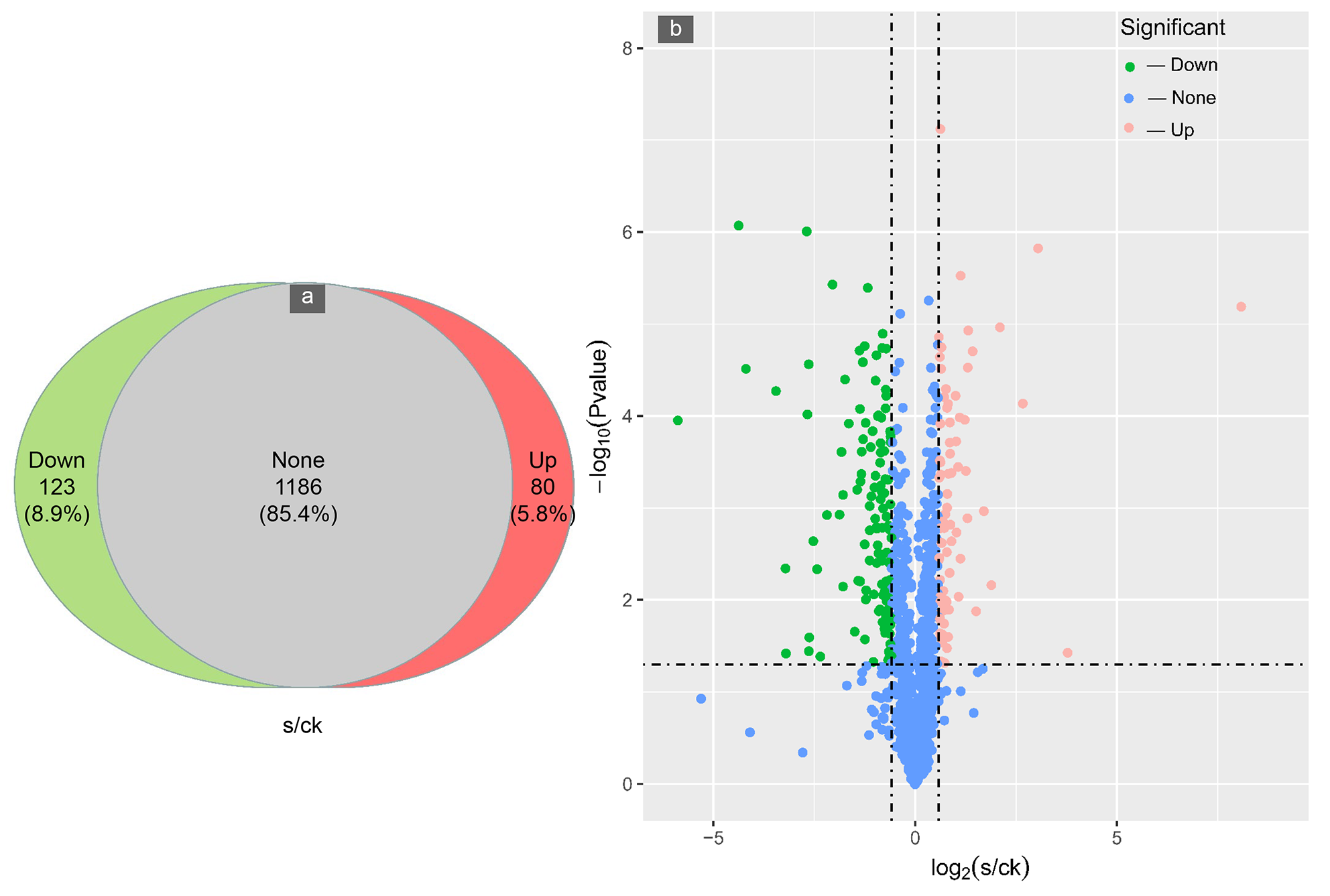

Supplement: S1 Fig — Venn diagrams (a) and Volcano plot (b) of expressed proteins between control and treatment groups. (TIF) [file pone.0250712.s001.tif]

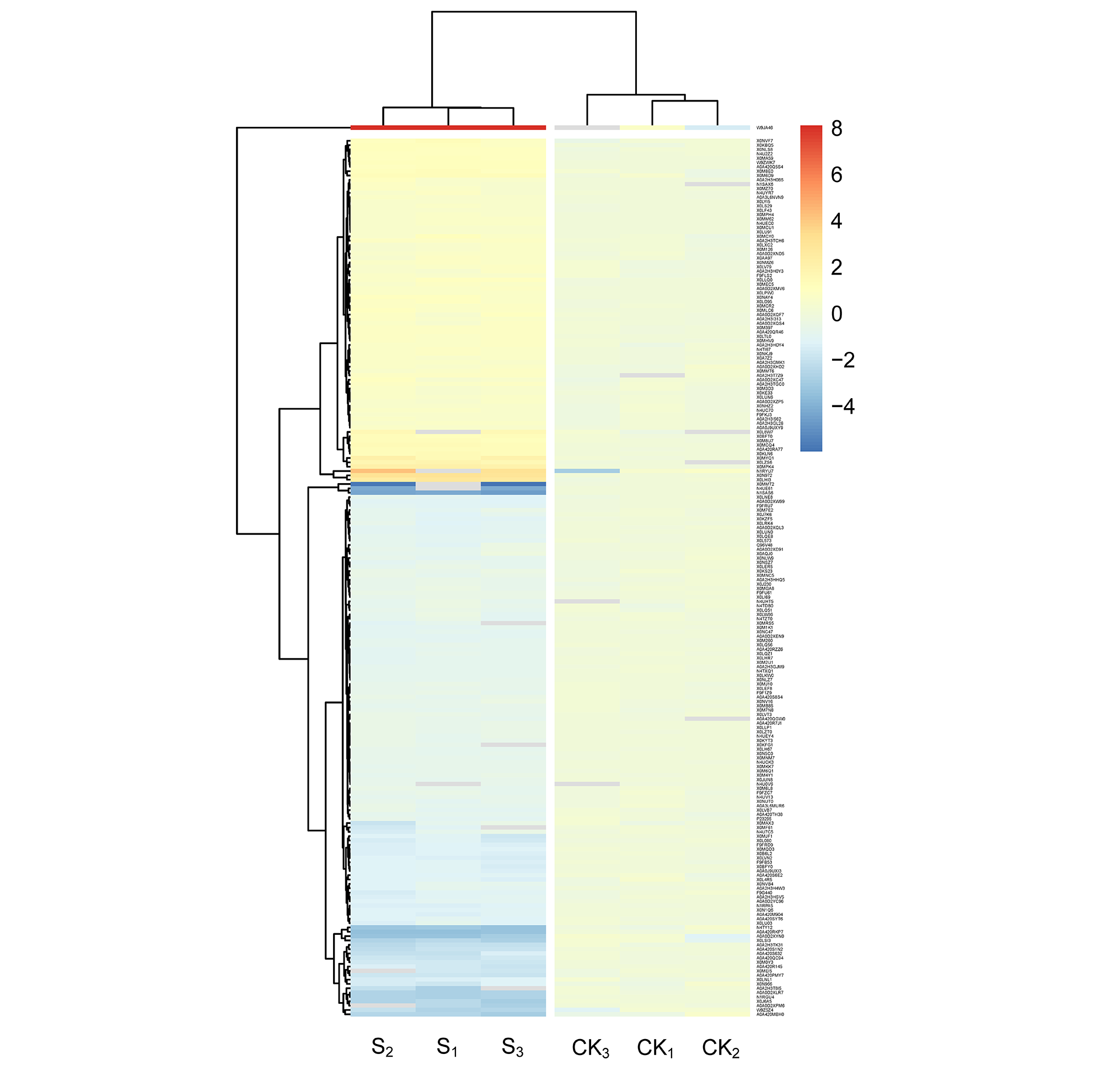

Supplement: S2 Fig — The relative abundance of proteins is represented by different colors, where red represents higher intensity and blue represents lower intensity. (TIF) [file pone.0250712.s002.tif]

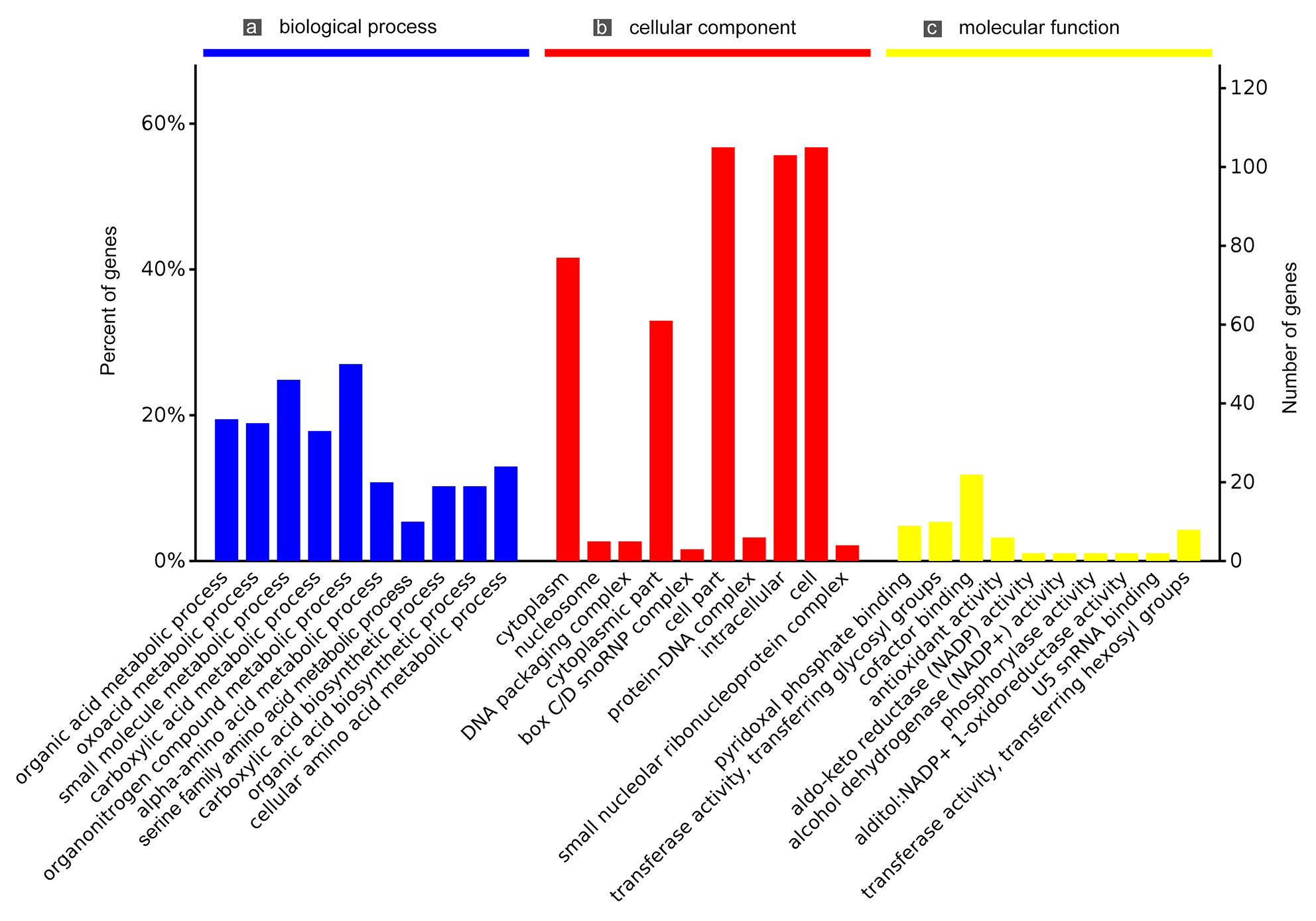

Supplement: S3 Fig — GO analysis of biological process (a), cellular component (b), and molecular function (c). (TIF) [file pone.0250712.s003.tif]

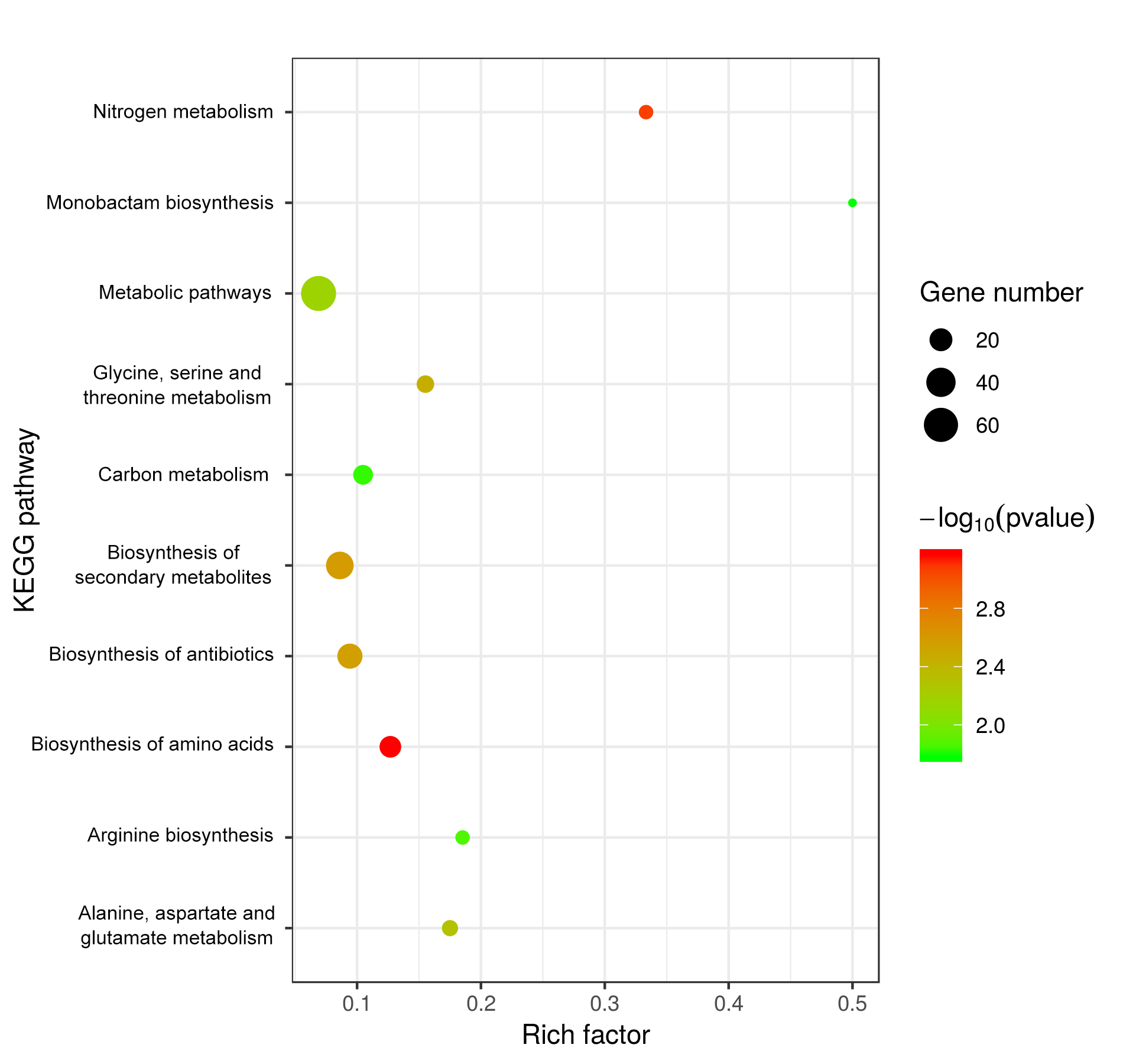

Supplement: S4 Fig — The vertical axis represents the enriched KEGG classification (fold > 1.5, p < 0.05). The horizontal axis is the rich factor (rich factor ≤ 1), representing the ratio of the number of differentially expressed proteins to those identified in the KEGG pathway. The size of the circular area represents the number of differentially expressed proteins, and the circular color represents the enrichment p-value of the differentially expressed proteins under the KEGG classification. (TIF) [file pone.0250712.s004.tif]
